# Research and practice priorities in pilonidal sinus disease: a consensus from the PITSTOP study

**DOI:** 10.1111/codi.16946

**Published:** 2024-04-26

**Authors:** Matthew J. Lee, Ellen Lee, Mike Bradburn, Daniel Hind, Emily B. Strong, Farhat Din, Arkadiusz P. Wysocki, Jon Lund, Christine Moffatt, Jonathan Morton, Asha Senapati, Helen Jones, Steven R. Brown, the PITSTOP Management Group and the PITSTOP Validators, Abidemi Adeosun, Assad Aghahoseini, Richard Brady, Graham Branagan, Dermot Burke, Chris Challand, Sanjay Chaudri, Julie Cornish, Emma Court, Melissa Cunha, Ian Daniels, Nicola Eardly, Ryan Edridge, Ian Farrell, Jennie Grainger, Athur Harikrishnan, Farzana Kausir, Krishan Kumar, Hans Lederhuber, Michael Lim, Corie MacDonald, Yasuko Maeda, Sunanda Mahapatra, Kit Matthews, Christine Moffatt, Jonathan Morton, Susan Moug, Margaret Ogden, Jack Ollerhead, James Pederson, Clair Phillips, Thomas Rippon, Franscois Runau, Asha Senapati, Oliver Shapter, Baljit Singh, Deb Smith, Ben Stubbs, Nicholas Symons, Greg Thomas, Chris Thompson, Jared Torkington, Christopher Van Dort, Jenna Welsh, Annabelle Williams, Catherine Winton, Neil Yeomans

**Affiliations:** ^1^ Department of Oncology and Metabolism, School of Clinical Medicine University of Sheffield Sheffield UK; ^2^ Academic Directorate of General Surgery Sheffield Teaching Hospitals NHS Foundation Trust Sheffield UK; ^3^ Sheffield Clinical Trials Research Unit, School of Health and Related Research (ScHARR) University of Sheffield Sheffield UK; ^4^ Academic Coloproctology, Institute of Genetics and Cancer University of Edinburgh, Western General Hospital Edinburgh UK; ^5^ Logan Hospital Brisbane Queensland Australia; ^6^ Royal Derby Hospital University Hospitals of Derby and Burton Derby UK; ^7^ Nottingham University Hospitals NHS Trust Nottingham UK; ^8^ Addenbrookes Hospital Cambridge University Hospitals Cambridge UK; ^9^ St Mark’s Hospital London UK; ^10^ Queen Alexandra Hospital Portsmouth UK; ^11^ Oxford University Hospitals NHS Foundation Trust Oxford UK

**Keywords:** Delphi, pilonidal sinus, policy, priority, research

## Abstract

**Aim:**

Pilonidal sinus disease is a common condition treated by colorectal surgeons. There is a lack of literature in the field to guide optimal management of this condition. As part of the PITSTOP study, we aimed to identify policy and research priorities to provide direction to the field.

**Method:**

Patients and surgeons were invited to participate. A ‘So what, now what’ exercise was conducted, informed by data from PITSTOP. This generated statements for research and practice priorities. A three‐round online Delphi study was conducted, ranking statements based on policy and research separately. Statements were rated 1 (not important) to 9 (important). Statements that were rated 7–9 by more than 70% of participants were entered into the consensus meeting. Personalized voting feedback was shown between rounds. A face‐to‐face meeting was held to discuss statements, and participants were asked to rank statements using a weighted choice vote.

**Results:**

Twenty‐two people participated in the focus group, generating 14 research and 19 policy statements. Statements were voted on by 56 participants in round 1, 53 in round 2 and 51 in round 3. A total of 15 policy statements and 19 research statements were discussed in the consensus round. Key policy statements addressed treatment strategies and intensity, surgeon training opportunities, need for classification and the impact of treatment on return to work. Research recommendations included design of future trials, methodology considerations and research questions.

**Conclusion:**

This study has identified research and policy priorities in pilonidal sinus disease which are relevant to patients and clinicians. These should inform practice and future research.

**What does this paper add to the literature?**

This paper highlights practice priorities to improve the care of people with pilonidal sinus disease. It proposes research priorities to be addressed in future studies on this condition.

## INTRODUCTION

Pilonidal sinus disease (PD) results from the ingrowing of hairs, usually in the natal cleft. It is common, affecting around 26/100 000 of the population [1] and represents a major burden on health services with more than 13 000 hospital admissions per year in the UK [2].Treatment is mainly surgical [3], but evidence from previous work suggests a high rate of complications [4] and wound morbidity from intervention [5]. As the condition tends to affect a predominantly young and economically active population, this morbidity and the subsequent delay in recovery can have a significant impact on society. Despite this societal burden, the literature to support practice is of poor quality and regularly fails to advance the field [6]. This is reflected in the marked heterogeneity in practice [4, 7].

The Pilonidal sinus Treatment: STudying the OPtions (PITSTOP) project was a multimethod study exploring practice in the management of PD in the UK [8]. Part of the brief for that study was to identify priorities for research and practice to guide development in the field, and to improve the care of patients. The aim of this study was to use data from the PITSTOP study to identify research and practice priorities with the support of clinicians and patients.

## METHOD

This consensus exercise was conducted as part of the PITSTOP project, a multimethod evaluation of current management of PD. The study received approval from East of England – Cambridge South Research Ethics Committee (REC reference 18/EE/0370).

### Stakeholders

Two stakeholder groups were defined: patients with previous experience of PD and clinicians with an interest in PD. Clinicians included those with completion of training (CCT) in general surgery and nurse specialists with wound care practice. Participants were recruited via email, national organizations and social media. Participants were invited to participate in one, two or all three phases of the consensus exercise.

Patient representatives with relevant experience were recruited to the patient stakeholder group following Delphi conception and contributed to the delivery and analysis across all three phases of the study. Reporting is in line with GRIPP2SF [9].

### Phase 1: item generation

In accordance with Delphi methodology, the study consisted of three phases. In phase 1, an online workshop was conducted. This was based on Rolfe's critical reflection model, ‘What? So what? Now what?’ [10]. In the ‘What?’ phase data or information are presented. In this case, researchers presented findings from the cohort study, mixed‐method study and survey‐based work. In the ‘So what?’ phase, participants are encouraged to reflect and discuss the information. Participants were asked to consider how the presented data reflected their experiences, and how this matched wider experiences. In the ‘Now what?’ phase, participants discussed how the information should be used to influence the next stage. Participants were asked to frame their ideas as statements related to policy or research ideas.

In the workshop, the following data were presented: preliminary PITSTOP cohort data, a systematic review of classification systems [11], a mapping review of PD [3], the PITSTOP discrete choice experiment survey [12] and the PITSTOP mixed‐methods study [5]. Participants were asked to consider two questions: ‘How can we use this data to improve and/or inform clinical practice?' and ‘What are the key research questions generated by this data?’. Participants were split into four equal groups to discuss these different data sources. Each group was facilitated by a clinical and nonclinical facilitator. Contemporaneous notes were taken using Google Jamboard (Palo Alto, CA) to capture statements and supporting context. A long list of potential practice and research recommendation statements was generated. The steering committee assessed the readability of these statements. Prior to attending the workshop, all participants received an information sheet and completed an online electronic consent form.

### Phase 2: modified eDelphi

In phase 2, a three‐round eDelphi consensus was conducted. The Delphi surveys were delivered using Qualtrics. In round 1, all participants were emailed a participant information sheet and a link to the survey. Upon accessing the survey, participants were asked to complete an online consent form. The following information was captured: age, gender, demographics, ethnicity, email address and stakeholder respondent group (patient, surgeon or specialist nurse). The long list of recommendation statements was presented in a random order and each statement was supplemented with a written summary to aid understanding. At the end of the survey, respondents were encouraged to propose any additional statements. Additional items were reviewed by the steering group at the end of round 1.

In rounds two and three, the remaining longlisted items were presented in random order. Ratings of items were reviewed after the close of each round. Respondents received an email copy of results which included their vote and how that compared with each stakeholder group's votes.

During each round, participants voted on the importance of each recommendation using a nine‐point Likert scale (1 being not important and 9 being very important). Recommendations were shortlisted if the following was satisfied: (1) >70% of participants within both stakeholder groups rate the recommendation as 7–9; (2) 90% of participants within a single stakeholder group rated an item 7‐9. Recommendations that reached consensus after three rounds were considered at the consensus meeting. All items had to be rated to complete the surveys. Only those who completed a survey round were eligible to participate in the subsequent round.

### Phase 3: consensus meeting

Participants were drawn from those who expressed an interest in attending the consensus exercise from the third round of the eDelphi. Efforts were made to recruit a minimum of 15 participants, with at least three patient representatives in attendance. Maximum attendance was set at 20 to maximize opportunities for participation. Participants provided written informed consent prior to participation.

A virtual meeting was held on the Google Meet platform. Participants were presented with policy items that had reached consensus. Following review, free discussion was invited for participants to highlight those they felt to be important, or to provide context. Participants were then asked to complete a ‘constant sum’ exercise on the Qualtrics platform. In this, they were asked to allocate all of 100 points to their key policy priorities. This allocation could be done in any combination as long as 100 points were allocated. The five items with the highest sum of points were allocated to them were a priority. This was then repeated for research priorities.

## RESULTS

### Phase 1: item generation

The item generation exercise was attended by 16 clinicians and six patients. Following this exercise, 33 items were generated for voting (Table 1).

**TABLE 1:** Longlist of items.

Policy statement	Research statement
Any treatment of pilonidal disease should aim to be less disruptive than the disease itself	A future randomized trial (RCT) in the treatment of pilonidal sinus should compare widely used techniques
Surgeons should have access to opportunities to learn new techniques for the treatment of pilonidal sinus disease	Postsurgical care (e.g. wound care, follow‐up etc.) is an important part of treatment strategy. Further work is required to establish the optimum way to deliver this
Lay open is associated with slow healing and delayed return to normal activities. It should rarely be considered as the first treatment option	Future research should aim to define an algorithm or decision tree to aid surgeon decision‐making
Minimally invasive techniques should be considered as the first‐line intervention, as these are associated with low operative morbidity and comparable recurrence and healing rates to more extensive interventions	A future randomized trial (RCT) should include two broad groups of interventions – major (i.e. asymmetric closure, leave open and midline closure) versus minor (i.e. minimal excision)
There is a need for a standard classification system/tool for pilonidal sinus disease	A decision aid targeted at patients to understand help treatment options might improve patient satisfaction with treatment
Any classification tool should be easy to use	Classification should include assessment of symptoms
A classification tool for pilonidal sinus should help to inform treatment options	Classification systems should include data related to hair type and distribution
Patients should be counselled about the risk of recurrence	Classification systems should include data on recurrent skin infections in nonpilonidal areas
Patients should be counselled about the impact of treatments on return to normal activities	Classification systems should include data on extent of disease beyond the natal cleft
Patients may wish for symptomatic improvement rather than cure, and this should be explored in early discussions	Consistency in reporting patient and disease factors would help us better understand what characteristics are associated with good or bad outcomes
Clinicians and researchers need to clearly define failure of healing versus recurrence as the two may present similarly	A core outcome set for pilonidal disease might help us understand what outcomes are important to clinicians and patients following treatment of pilonidal disease. It may also improve future evaluations of treatments
Delayed return to work is an important outcome following treatment	There is a need for a patient‐reported outcome to be used in future pilonidal sinus research
A tool is needed to measure the impact of treatments/disease on quality of life (e.g. a disease‐specific patient‐reported outcome measure)	Future research should explore whether hair removal reduces the risk of wound complications or recurrence of pilonidal disease
We need to determine how long we should wait before deciding that wound healing is delayed or failed	Future research should explore whether weight loss reduces the risk of wound complications or recurrence of pilonidal disease
Follow‐up should continue until there is evidence of complete wound healing	Future research should explore whether smoking behaviours reduces the risk of wound complications and/or recurrence of pilonidal disease
Patients with symptomatic pilonidal disease always require a secondary care referral	Future research should assess the role of postoperative antibiotic treatment in wound healing and/or recurrence
Novel minimally invasive procedures (e.g. laser) should be thoroughly appraised in randomized trials before general adoption	Future research should explore the role wound dressings play in wound healing and/or recurrence
Imaging is rarely useful in pilonidal disease	A future randomized trial (RCT) should compare procedures in mild or minimal disease where the wound is left open (e.g. pit picking and EPSiT) versus closure of the wound (e.g. glue)
Shared decision‐making should be employed when discussing treatment options	A future randomized trial (RCT) should compare nonexcisional therapies
	Future research should explore the role of patient characteristics including genetics and microbiome on the pilonidal disease process
	Wide excision and leave open procedures should not be included in any future trial
	Future research should compare major procedures (e.g. flaps) against minor procedures (e.g. pit picking, glue) stratified by disease severity

Abbreviation: EPSiT, endoscopic pilonidal sinus treatment.

### Phase 2: Delphi voting

**FIGURE 1:**
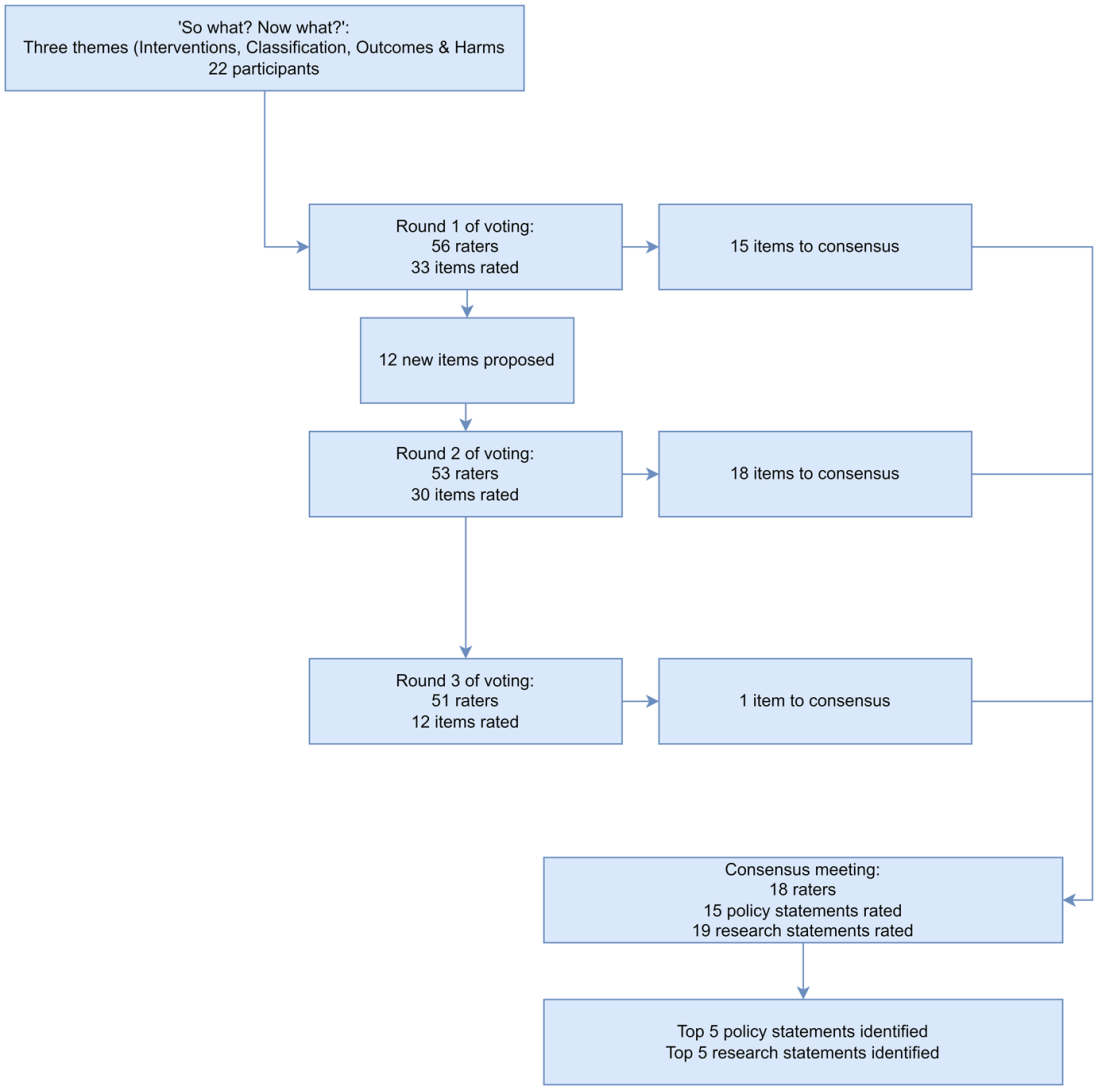
Flow of items through Delphi.

In round 1, 56 participants voted on 33 items. Fifteen items met the inclusion criteria and were passed to the consensus meeting. A further 12 items were proposed for round 2. In round 2, 53 participants rated 30 items, and 18 met the inclusion criteria. Finally, in round 3, 51 raters voted on 12 items and one further item met consensus. The flow of items can be seen in Figure 1, and participant characteristics in Table 2. Voting outcomes on included items are shown in Table S1.

**TABLE 2:** Participants in Delphi.

		Round 1	Round 2	Round 3
Participant type	Patient	15	14	14
	Surgeon	40	38	36
	Nurse specialist	1	1	1
Retention rate	–	–	95%	91%

### Phase 3: consensus meeting

The consensus meeting was attended by three patient representatives and 15 clinicians. One clinician withdrew during the meeting due to work commitments. Participants were presented with a total of 34 statements – 15 policy and 19 research. After discussion and voting, five policy and five research statements were prioritized (Table 3). Policy statements addressed the use of minimally invasive procedures, avoidance of morbidity from treatment, training of surgeons, classification of disease and the importance of return to work as an outcome metric. For research statements, recommendations were made on pragmatic and explanatory trial designs, the need for a patient‐reported outcome measure (PROM) and the need for a core outcome set.

**TABLE 3:** Final prioritized items.

Statement number	Policy statement	Score
1	Any treatment of pilonidal disease should aim to be less disruptive than the disease itself	270
2	Minimally invasive techniques should be considered as the first‐line intervention, as these are associated with low operative morbidity and comparable recurrence and healing rates to more extensive interventions	174
3	Surgeons should have access to opportunities to learn new techniques for the treatment of pilonidal sinus disease	140
4	A classification tool for pilonidal sinus should help to inform treatment options	140
5	Delayed return to work is an important outcome following treatment	134

Statement number	Research statement	Score
1	A future randomized trial (RCT) should include two broad groups of interventions – major (i.e. asymmetric closure, leave open and midline closure) versus minor (i.e. minimal excision)	189
2	A core outcome set for pilonidal disease might help us understand what outcomes are important to clinicians and patients following treatment of pilonidal disease. It may also improve future evaluations of treatments	179
3	Future research should compare major procedures (e.g. flaps) against minor procedures (e.g. pit picking, glue) stratified by disease severity	148
4	There is a need for a patient‐reported outcome to be used in future pilonidal sinus research	119
5	Future research should aim to define an algorithm or decision tree to aid surgeon decision‐making	100

### Public and Patient Involvement (PPI) feedback

Fifteen patient representatives with relevant experience were recruited to the patient stakeholder group following substudy conception and contributed to the delivery and analysis. Six of 15 PPI representatives attended the initial workshop and supported the generation of the long list of recommendations. Four of 15 PPI representatives attended the virtual consensus meeting and highlighted the importance of ensuring that the final set of recommendation statements were conceivable to a patient audience.

## DISCUSSION

This priority‐setting exercise has identified a series of policy and research statements that can be immediately acted upon by the surgical community. These consensus statements provide a foundation for the necessary improvement of research into the care of those with PD.

All the policy recommendations are in a way complimentary. The recommendation with by far the highest sum score recommends efforts to ensure a treatment does not make the patient's condition worse than their baseline. This statement is consistent with the second recommendation that minimally invasive, skin‐preserving approaches should be considered more frequently. These recommendations draw directly from the PITSTOP cohort [4], mixed‐method studies of regret [5] and discrete choice experiments [12]. Data from the cohort study show that complications are more likely with more major skin excisional approaches and recovery is longer. Whilst treatment failure and recurrence are lower, data from the discrete choice experiment suggests that many patients would trade this higher risk of failure for more rapid recovery. This is consistent with the fifth policy recommendation, which stresses return to work as a key metric when considering the surgical approach.

One of the reasons why some surgeons do not offer minimally invasive techniques is lack of training. Our survey of practice suggests that many surgeons favour one ‘go to’ operation and do not have the training to vary the approach according to the degree of disease and patient outcome preference [7]. Specific technical requirements for PD are absent from the current UK training programme [7]. Training relies mainly on the preferences of the trainers, leading to the perpetuation of older, possibly obsolete, interventions. Access to opportunities to learn newer techniques is highlighted in the third policy recommendation.

If an armamentarium of approaches for PD are to be recommended there is a need to classify the disease, potentially helping the surgeon and patient to decide the optimum approach for the desired outcome. Several such tools exist for PD, although none are in mainstream use [11]. Work from PITSTOP demonstrated the properties of the International PIlonidal Sinus (Berlin) classification [13, 14], and this may be an appropriate tool to use in clinical settings.

The research recommendations provide future direction on methodology and study design. One difficulty with study design and utilization of a randomized controlled trial is the large number of potential interventions with no obvious gold standard procedure. A pragmatic approach would be to group interventions with similar underlying principles. The broadest grouping involves those interventions aimed at preserving tissue (minimally invasive techniques such as pit picking, curettage and glue, endoscopic pilonidal sinus treatment, etc.) and those resulting in more major skin excision with or without defect closure (Karydakis, Bascom cleft closure, flap procedures etc.). Such a trial could allow the surgeon to select their favoured procedure from each group to deliver a ‘dealer's choice’. This is similar to the FIAT trial that allowed surgeons to choose the procedure delivered in the control arm [15]. This makes for a more achievable trial in a multicentre setting, particularly given variation of practice in the UK.

Recommendation 2 involves the development of a core outcome set [16]. This is a widely accepted methodology which encourages patients and clinicians to agree on a common set of outcomes to be reported in all future studies on a condition. Such sets have been developed for other surgical conditions [17, 18]. Participants also felt that the development of a disease‐specific PROM should be a priority. Whilst generic measures such as EQ‐5D‐5 L and SF‐36 are widely used to provide an overview of quality of life they may not capture key symptoms relevant to the condition being treated [19, 20]. Given the location of PD, and the potential wound problems, disease‐specific questions might address pain, discharge and wound problems not captured elsewhere.

The final statement recommended that research should aim to define an algorithm or decision tree to aid decision‐making. There are two potential methods of delivering this recommendation. First, a multiarm, multistage trial (MAMS) allows randomization at different phases of treatment, for instance first presentation, second presentation, etc. [21]. While this requires significant resources to design and deliver, it would concentrate PD research into a single large study and potentially drive a step change in disease management by addressing treatment at different stages and presentations. The alternative approach is to establish a large cohort study using matching approaches as observed in the PITSTOP cohort study, and use clinical and outcome data to support the development of a decision aid, similar to that demonstrated in the AGE‐GAP study in breast cancer [22].

There are limitations to this consensus. There were 65 participants in round 1, many of whom were involved in the PITSTOP study. Results may therefore reflect the opinions of enthusiasts rather than generalists. The study did, however, take a robust methodological approach. Item generation was structured and based on recent real‐world data. Iterative voting was undertaken with minimal attrition and patient participation at all stages. Finally, the consensus statements are broad and easy to convert into practice. In particular, the research recommendations highlight methodology issues needed to improve on the current poor standard of PD research [5].

Impacts of this research are clear. Clinicians and commissioners of services should implement policy recommendations into their standard operating procedures and guidance. Relevant speciality associations should consider methods for delivering additional training for surgeons, potentially supported by industry. Routine metrics should be collected for services including rates of minimally invasive techniques and time to return to work. Research funders and researchers should consider action on the methodological aspects of the research recommendations first and, once these are addressed, support the delivery of a major trial or cohort study.

## AUTHOR CONTRIBUTIONS

**M. J. Lee:** Conceptualization; investigation; funding acquisition; writing – original draft; methodology; writing – review and editing; formal analysis; supervision; project administration; software. **E. B. Strong:** Investigation; writing – original draft; methodology; writing – review and editing; formal analysis; project administration; data curation. **D. Hind:** Conceptualization; investigation; funding acquisition; writing – original draft; methodology; formal analysis; project administration; supervision; writing – review and editing. **H. Jones:** Conceptualization; investigation; writing – original draft; methodology; writing – review and editing; project administration. **S. R. Brown:** Conceptualization; investigation; funding acquisition; writing – original draft; validation; methodology; writing – review and editing; supervision.

## CONFLICT OF INTEREST STATEMENT

None to declare.

## ETHICS STATEMENT

The study received approval from East of England—Cambridge South Research Ethics Committee (REC reference 18/EE/0370).

## Supporting information


Table S1

## Data Availability

The data that supports the findings of this study are available in the supplementary material of this article.
