# Supplementary material for: Research and practice priorities in pilonidal sinus disease: a consensus from the PITSTOP study
Source: Colorectal Dis. 2024 Apr 26;27(1):e16946. doi: 10.1111/codi.16946 (PMC11683161; doi:10.1111/codi.16946)
Supplement: Supplementary file 1 — Table S1 [file CODI-27-0-s001.docx]

Supplementary material

S1. Ratings presented by statement within each stakeholder group.

|  | **Round 1** | | | **Round 2** | | | **Round 3** | | |
| --- | --- | --- | --- | --- | --- | --- | --- | --- | --- |
| **Statement** | **Clinicians** | **Patients** | **Both** | **Clinicians** | **Patients** | **Both** | **Clinicians** | **Patients** | **Both** |
| Any treatment of pilonidal disease should aim to be less disruptive than the disease itself. | 82.5% | 80.0% | 81.8% | To consensus | | | | | |
| Surgeons should have access to opportunities to learn new techniques for the treatment of pilonidal sinus disease. | 97.5% | 73.3% | 90.9% | To consensus | | | | | |
| Lay open is associated with slow healing and delayed return to normal activities. It should rarely be considered as the first treatment option. | 60.0% | 60.0% | 60.0% | 60.5% | 76.9% | 64.7% | 62.2% | 78.6% | 66.7% |
| Minimally invasive techniques should be considered as the first line intervention, as these are associated with low operative morbidity and comparable recurrence and healing rates to more extensive interventions. | 65% | 86.7% | 70.9% | 68.4% | 84.6% | 72.5% | To consensus | | |
| There is a need for a standard classification system/tool for pilonidal sinus disease. | 82.5% | 53.3% | 74.5% | 81.6% | 84.6% | 82.4% | To consensus | | |
| Any classification tool should be easy to use. | 92.5% | 46.7% | 80.0% | To consensus | | | | | |
| A classification tool for pilonidal sinus should help to inform treatment options. | 82.5% | 66.7% | 78.2% | 76.3% | 92.3% | 80.4% | To consensus | | |
| Patients should be counselled about the risk of recurrence. | 97.5% | 80.0% | 92.7% | To consensus | | | | | |
| Patients should be counselled about the impact of treatments on return to normal activities. | 95% | 80.0% | 90.9% | To consensus | | | | | |
| Patients may wish for symptomatic improvement rather than cure, and this should be explored in early discussions. | 80.0% | 53.3% | 72.7% | 84.2% | 69.2% | 80.4% | To consensus | | |
| Clinicians and researchers need to clearly define failure of healing vs recurrence as the two may present similarly. | 57.5% | 80.0% | 63.6% | 73.7% | 92.3% | 78.4% | To consensus | | |
| Delayed return to work is an important outcome following treatment. | 90% | 73.3% | 85.5% | To consensus | | | | | |
| A tool is needed to measure the impact of treatments/disease on quality of life (e.g. a disease specific patient reported outcome measure). | 82.5% | 60.0% | 76.4% | 84.2% | 69.2% | 80.4% | To consensus | | |
| We need to determine how long we should wait before deciding wound healing is delayed or failed. | 45% | 60.0% | 49.1% | 60.5% | 76.9% | 64.7% | 62.3% | 71.4% | 64.7% |
| A future randomised trial (RCT) in the treatment of pilonidal sinus should compare widely used techniques. | 90% | 53.3% | 80.0% | To consensus | | | | | |
| Post-surgical care (e.g., wound care, follow-up etc.) is an important part of treatment strategy. Further work is required to establish the optimum way to deliver this. | 87.5% | 73.3% | 83.6% | To consensus | | | | | |
| Future research should aim to define an algorithm or decision tree to aid surgeon decision making. | 77.5% | 80.0% | 78.2% | To consensus | | | | | |
| A future randomised trial (RCT) should include two broad groups of interventions - major (i.e., asymmetric closure, leave open and midline closure) versus minor (i.e., minimal excision). | 67.5% | 60.0% | 65.5% | 71.1% | 92.3% | 76.5% | To consensus | | |
| A decision aid targeted at patients to understand help treatment options might improve patient satisfaction with treatment. | 80.0% | 80.0% | 80.0% | To consensus | | | | | |
| Classification should include an assessment of symptoms. | 82.5% | 60.0% | 76.4% | 89.5% | 92.3% | 90.2% | To consensus | | |
| Classification systems should include data related to hair type and distribution. | 32.5% | 40.0% | 34.5% | 50.0% | 76.9% | 56.9% | 51.4% | 57.1% | 52.9% |
| Classification systems should include data on recurrent skin infections in non-pilonidal areas. | 45.0% | 66.7% | 50.9% | 44.7% | 30.8% | 41.2% | 54.1% | 42.9% | 51.0% |
| Classification systems should include data on extent of disease beyond the natal cleft. | 85.0% | 46.7% | 74.5% | 81.6% | 46.2% | 72.5% | To consensus | | |
| Consistency in reporting patient and disease factors would help us better understand what characteristics are associated with good or bad outcomes. | 85.0% | 66.7% | 80.0% | 84.2% | 92.3% | 86.3% | To consensus | | |
| A core outcome set for pilonidal disease might help us understand what outcomes are important to clinicians and patients following treatment of pilonidal disease. It may also improve future evaluations of treatments. | 95.0% | 53.3% | 83.6% | To consensus | | | | | |
| There is a need for a patient reported outcome to be used in future pilonidal sinus research. | 90.0% | 86.7% | 89.1% | To consensus | | | | | |
| Future research should explore whether hair removal reduces the risk of wound complications or recurrence of pilonidal disease. | 90.0% | 86.7% | 89.1% | To consensus | | | | | |
| Future research should explore whether weight loss reduces the risk of wound complications or recurrence of pilonidal disease. | 52.5% | 53.3% | 52.7% | 57.9% | 53.8% | 56.9% | 56.8% | 42.9% | 52.9% |
| Future research should explore whether smoking behaviours reduces the risk of wound complications and/or recurrence of pilonidal disease. | 92.5% | 46.7% | 80.0% | To consensus | | | | | |
| Future research should assess the role of post-operative antibiotic treatment in wound healing and/or recurrence. | 70.0% | 66.7% | 69.1% | 60.5% | 92.3% | 68.6% | To consensus | | |
| Future research should explore the role wound dressings play in wound healing and/or recurrence. | 75% | 60.0% | 70.9% | 60.5% | 76.9% | 64.7% | 73.0% | 78.6% | 74.5% |
| A future randomised trial (RCT) should compare procedures in mild or minimal disease where the wound is left open (e.g. pit picking and EPSiT) versus closure of the wound (e.g. glue). | 75% | 80.0% | 76.4% | To consensus | | | | | |
| A future randomised trial (RCT) should compare non-excisional therapies. | 77.5% | 60.0% | 72.7% | 81.6% | 76.9% | 80.4% | To consensus | | |
| Follow-up should continue until there is evidence of complete wound healing. |  |  |  | 60.5% | 92.3% | 68.6% | To consensus | | |
| Patients with symptomatic pilonidal disease always require a secondary care referral. |  |  |  | 55.3% | 69.2% | 58.8% | 59.5% | 57.1% | 58.8% |
| Novel minimally invasive procedures (e.g. laser) should be thoroughly appraised in randomised trials before general adoption. |  |  |  | 73.7% | 53.8% | 70.6% | To consensus | | |
| Imaging is rarely useful in pilonidal disease. |  |  |  | 42.1% | 30.8% | 39.2% | 32.4% | 42.9% | 35.3% |
| Shared decision making should be employed when discussing treatment options. |  |  |  | 86.8% | 92.3% | 88.2% | To consensus | | |
| Future research should explore the role of patient characteristics including genetics and microbiome on the pilonidal disease process. |  |  |  | 34.2% | 76.9% | 45.1% | 48.6% | 50.0% | 49.0% |
| Wide excision and leave open procedures should not be included in any future trial. |  |  |  | 44.7% | 38.5% | 43.1% | 45.9% | 42.9% | 45.1% |
| Future research should compare major procedures (e.g. flaps) against minor procedures (e.g. pit picking, glue) stratified by disease severity. |  |  |  | 76.3% | 92.3% | 80.4% | To consensus | | |
| Future research should explore the role of antibiotics in primary care management. |  |  |  | 34.2% | 84.6% | 43.1% | 35.1% | 71.4% | 45.1% |
| Future research should explore the role of antibiotics in secondary care management |  |  |  | 47.4% | 84.6% | 47.1% | 43.2% | 57.1% | 47.1% |
| Future research should include robust economic analysis. |  |  |  | 81.6% | 46.2% | 72.5% | To consensus | | |
| Future research should include decision regret as an outcome measure. |  |  |  | 73.7% | 61.5% | 70.6% | To consensus | | |
